# An overview of financial sources being utilized to support Zika Virus published research

**DOI:** 10.1371/journal.pone.0183134

**Published:** 2017-08-17

**Authors:** Keisha Goodridge, Ludovic Reveiz, Vanessa Elias

**Affiliations:** Knowledge Management, Bioethics and Research Office, Pan American Health Organization, Washington D.C., United States of America; Universidade de Mogi das Cruzes, BRAZIL

## Abstract

**Background:**

Since its initial detection in Brazil in 2015, Zika Virus (ZIKV) has spread rapidly throughout most of the Caribbean and South, Central and North America. An upsurge in congenital syndrome associated with ZIKV and Guillain-Barre Syndrome (GBS) has been associated with the increase in ZIKV. This amplification in numbers led to the need for funded research initiatives focusing on various countries globally and on specific experimental types.

**Objectives:**

To determine the financial institutions involved in the production of primary research into the ZIKV epidemic. This research also intends to draw attention to the investigative areas that are dominating, experimental types being conducted and the geographical areas that are producing the bulk of the research utilizing available funds.

**Methods:**

A cross sectional search of published primary research was conducted using Pan American Health Organization (PAHO) Zika platform and PubMed between January 2007 and October 2016. Titles, abstract and full articles were assed and one researcher extracted data. Information was crossed checked by a second researcher to ensure accuracy.

**Findings:**

268 articles were included and investigations occurred across 48 countries with Brazil and USA dominating the research. Applied Research and Laboratory based studies were most frequently utilized. 38.1% of articles did not report financial sources. Public institutions were the major known contributors. Other financiers included private, non-profits and mixed funders exclusive of public sources. 156 individual financial bodies assisted with National Institute of Health being most frequently mentioned followed by The National Council for Scientific and Technological Development (CNPq) and the Institut Pasteur. Virus, vectors and reservoirs was most frequently used (99/268, 36.9%) followed by clinical management (70/268, 26.1%) and epidemiology (46/268, 17.2%).

**Interpretation:**

The evidence suggests international efforts to fund ZIKV research and a need to foster collaborative and synchronized priority setting for resource allocation.

## Introduction

As of November 2016 autochthonous transmission of Zika Virus (ZIKV) has been reported in 48 countries and territories in the Americas [1]. ZIKV has spread rapidly throughout most of South America, Central America, North America and the Caribbean territories since its initial detection in Brazil in 2015 [1]. According the Pan American Health Organization (PAHO), confirmed cases of congenital syndrome associated with ZIKV infection have been reported in 22 countries and territories in the Americas [1] Additionally, 13 Regions reported an increase in Guillain-Barre syndrome (GBS) cases with laboratory-confirmation of ZIKV in at least one of the cases [1]. This increase in incidences led the PAHO issuing an epidemiological alter on May 7^th^ 2015 declaring ZIKV an international emergency and requesting research into transmission, surveillance, laboratory detection and case management [2]

Since the 2007 outbreak of the ZIKV in Asia and the 2015 outbreak in Brazil, there has been a steady increase in ZIKV research to answer research questions related to virus control, transmission, clinical management and spread. This increase has been met with the production of research agendas and strategic plans by international health agencies as well as the donation and allocation of funds to be utilized in the ZIKV epidemic [3,4]. These agendas and plans focus on areas related to prevention and control, research and the identification of research gaps. The Global Health Network highlights some of the funding initiatives for research projects related to ZIKV[5] Organizations such as the European Commissions: Horizon 2020, the US National Institute of Health, The United States Department of Defense, national and federal Brazilian agencies among many others aim to address research gaps associated with the ZIKV and other emerging threats in Latin American and Caribbean (LAC) countries and worldwide [5,6].

Estimates have been made regarding the amount of economic resources that will be required to fund the current ZIKV outbreaks. The World Health Organization (WHO) has established a Strategic Response Framework and Joint Operations Plan with its partners which has suggested that $56 million USD would be required to focus on areas such as research, surveillance and response for an established period [7] These partners include bodies such as AmeriCares, United Nations Development Program (UNDP), World Vision and UN Women [7]. This framework has indicated that although several organizations have stated their intent to fund the ZIKV initiatives, there still remains the question as to the definite amount of resources that are received and to whom these funds are allocated. Interim documentation on the global response to ZIKV provided by WHO has shown that only portions of requested funds have been received by WHO and its partners[7]. The UNDP for example has requested over 4 million dollars in funding but have only received a portion of these monies [7].

This information thus suggests that funds are in fact being sourced to combat ZIKV. However, in order to ensure these available resources are allocated in a manner to maximize priority areas, up to date and accurate information is needed on financial needs and distribution. The purpose of this article is thus to highlight the present financial climate as it relates to ZIKV research by examining: (i) which organizations are financial supporters of ZIKV research (ii) experimental types and procedures being investigated with available funds and (iii) which are the countries and geographical regions obtaining ZIKV research financial incentives. This information will give all parties involved the opportunity to make better coordinated decisions in the future as to where ZIKV research needs to go in terms of funding distribution, research study design and locations where there is a need for increased research.

## Methods

### Objective

To provide up to date information on the type of organizations responsible for funding ZIKV research, the countries that receive this funding and the study designs being utilized in order to determine areas for additional support.

### Study design

A cross sectional study of primary research was performed using Pan American Health Organization (PAHO) (http://www.paho.org/zika-research/index.php/1/entry?search_form_id=1) and PubMed (https://www.ncbi.nlm.nih.gov/pubmed) databases. PAHO database compiles information on ZIKV protocols, research and epidemiological alerts from databanks such as Science Direct and Springer and journals such as PLOS Neglected Tropical Disease and Oxford Journals. All sources were searched independently by one researcher from September 26—October 13^th^ 2016.

### Search strategy

The PAHO Platform periodically searches the following databases and governmental websites: International Clinical Trials Registry Platform (ICTRP), Sistema Nacional de Ética em Pesquisa (SISNEP), Sistema de Informação da Rede Brasileira de Avaliação de Tecnologias em Saúde (SISREBRATS), Pesquisa Saúde (Pesquisa SUS), Conselho Nacional de Desenvolvimento Científico e Tecnológico (CNPq), Comissão da Coordenação de Aperfeiçoamento de Pessoal de Nível Superior CAPES, Ministério da Ciência, Tecnologia e Inovação, Registro peruano de ensayos clínicos y de estudios observacionales, Registro nacional de investigaciones en salud (RENIS), Registro nacional de ensayos clínicos (RNECCOFEPRIS); PAHO country offices and key national institutions are also periodically contacted to request access to information on published or unpublished study protocols. The PAHO Platform incorporates only primary research studies and case reports or case series. The articles retrieved in this search were reviewed and those published from January 2007 to September 2016 were included. For the ‘Type of Publication’ selection criteria, only Published Articles were chosen. All General Categories provided by the PAHO Platform (Public Health Interventions, Causality, Clinical Management, Epidemiology, Virus Vectors and Reservoirs, Disease pathogenesis and consequences of ZIKA infection, Health System and Service response, Research and development of products) were included in this search. Given the periodicity of update of the PAHO database, Pubmed; Lilacs and Embase were searched using the following terms (Zika [mh] OR Zika [tw] OR Zika virus [mh] OR Zika virus [tw]). This additional search was conducted to locate articles published from 1st August 2016 to 10th October 2016 that may not have been retrieved using PAHO Platform (given the periodicity of updates).

### Inclusion criteria

The following inclusion criteria were utilized: (1) articles on primary studies investigating ZIKV published from January 2007 to October 2016; (2) full text availability and (3) articles published in English language. No restriction was applied based on study design and study participant type. The search identified a total of 585 references of which 268 met the inclusion criteria.

### Data extraction

Data was extracted by one independent researcher utilizing a pre-constructed data extraction form which collected information on: reference, country where study was conducted, funding organization type, location of funding body and type of study design. Complied data was then re-checked by a second researcher to ensure accuracy. A web search on funding organization specifications was conducted to verify information on funding organization type and location. The types of funding organization were categorized as: non-profit, public, private, non-governmental, mixed and not reported. Categories unknown and none/not specified were used to categorize references where type of funding institution could not be verified and references that reported that they received no funding respectively.

### Study analysis

Descriptive analysis was utilized to present the information on the types of organization responsible for funding ZIKV research, the countries which received this funding and the types of study designs being utilized with these available resources. Chi-square (X^2^) statistics were used to examine association between the above mentioned variables. A review protocol for this manuscript does not exist.

## Results

**Fig 1:**
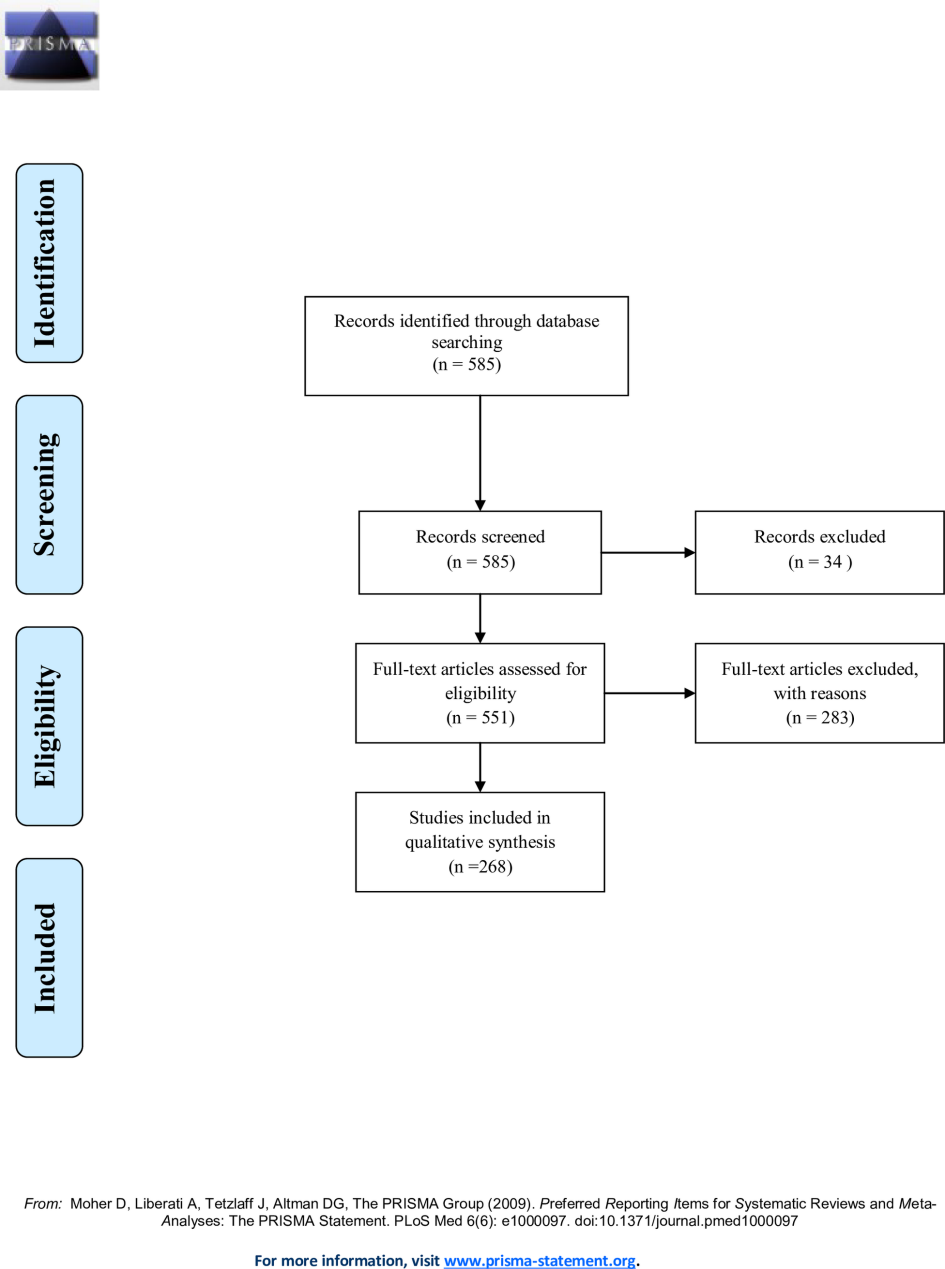
PRISMA flow diagram of studies included in research into Zika virus funding.

The search returned a total of 585 results of which 268 met the inclusion criteria (**Fig 1**). In the end, the primary researching country in the included articles was represented by 48 nations with Brazil (50/268, 18.7%) and USA (58/268, 21.6%) dominating the research. Other nations participating in ZIKV research include Latin American and the Caribbean (exclusive of Brazil) (23/268, 8.6%), China (20/268, 7.5%) and France (15/268, 5.6%). Of the 268 studies, 25/268, 9.3%, reported collaborating with one additional country for research. Of the twenty-five collaborating countries, 16/25, 64% reported collaboration with 3 countries and 8/25, 32% collaborated with three or more countries.

Study participation in the included studies comprised of adults, children and animals. The most frequently used category was described as other (68/268, 25.4%) and included studies that utilized cells and genes from the ZIKV, stem cells and other cellular bodies. Other categories consisted of adults only: females (both pregnant and not) and males (39/268, 14.6%); animals (36/268, 13.4%) such as rats and mosquitoes; women only (34/268, 12.7%) and general (children and adults of both genders) (32/268, 11.9%).

Various areas of research were described among the included studies (**Table 1**). The most frequent of this was virus, vectors and reservoirs (99/268, 36.9%). Clinical management (70/268, 26.1%) and epidemiological (46/268, 17.2%) studies were the next most frequently used area of research. The least utilized area of research was causality (6/268, 2.2%). We have conducted a breakdown of the characteristics of the included studies (**S1 Appendix**)

**Table 1:** Frequency distribution of area of research utilized in Zika virus research

Area of research according to PAHO Regional Research Agenda	Frequency	Percentage (%)
Causality	6	2.2
Clinical Management	70	26.1
Disease Pathogenesis	24	9
Epidemiology	46	17.2
Public Health Interventions	10	3.7
Research and Development of Products	13	4.9
Virus, Vectors and Reservoirs	99	36.9
**Total**	**268**	**100**

Included studies employed a variety of study designs. Laboratory based research (80/268, 29.9%) was the most prevalent followed by case reports (68/268, 25.4%) and case series (54/268, 20.1%). Economic and validation studies both had frequencies of 1/268, 0.4%.

Overall, applied research represented 171/268, 63.8% of the investigations into ZIKV. Within this category, case series (50/171, 29.2%), case report (63/171, 36.8%) and laboratory studies (15/171, 8.8%) were utilized in the above mentioned frequencies. Basic research represented 90/268, 33.6% of the examinations and were vastly laboratory based (58/90, 64.4%). Experimental development (7/268, 2.6%) was the least frequently used research type and encompassed solely laboratory based studies.

A total of 102/268, 38.1% of the articles did not report sources of funding. An additional 19/268, 7.1% reported none/not specified financing and 13/268, 4.9% reported funding from sources that were not able to be verified as belonging to one of the funding categorizes. Therefore, 134/268, 50.0% of the research funding was known for this article. Public funding represented 126/134, 94.0% and included research that received mixed funding inclusive of a public entity. Non-profit funding was seen in 5/134 of the included articles while mixed funding that did not included a public source was seen in 1/134 of the included articles. Exclusively private funding occurred in 2/134 included studies in this review.

For the articles with known sources of funding, public sources are predominant across three types of research (applied, basic and experimental). Applied research receives the bulk of financing (69/134, 51.5%) which is primarily utilized by case reports and case series investigations (30/69, 43.5%). Experimental development receives financial assistance from exclusively public sources (4/134, 3.0%) and it focuses on laboratory based research. Basic research receives 61/134, 45.5% of the overall funding.

A total of 156 funding bodies are represented across all known sources of financial assistance for ZIKV research. USA located National Institute of Health (NIH) is the most frequently mentioned financial assisting body (15.9%). The Brazilian based National Council for Scientific and Technological Development (CNPq) is the second most frequent supplier of funds for ZIKV research (3.9%) followed by the Institut Pasteur of France (3.2%). The European Union (2.9%) and the Ministry of Science and Technology of China (2.6%) also have been found to frequently fund ZIKV research. Other Brazilian based bodies funding research include Sao Paulo Research Foundation (FAPESP) (2.6%), The Higher Education Personnel Improvement Coordination (CAPES) of the Ministry of Education (MEC) (2.3%) and the Foundation Carlos Chagas Filho Research Support of the State of Rio de Janeiro (FAPERJ) (2.3%). A total of 119 funding bodies (38.6%) were found to support ZIKV research only once across the included studies. We have displayed the frequency with which specific institutions fund ZIKV research (**S2 Appendix**).

Primary researching countries were ordered based on economic status to be: high income 166/268 (61.9%) middle income 94/268 (35.1%) and low income 8/268 (3.0%) countries. Low and middle income countries receive no funding from other sources other than public. High income countries are receiving most of the funding overall (57.5%) while low income countries receive the least (5.2%).

Of the 102 studies that do not report funding, 75/102, 73.5% are from high income countries and 25.5% are from middle-income countries. Low income countries represent 2.6% of the countries doing investigations but 87.5% of them do not report who supports the research.

## Discussion

This article recognized types of research and study designs that are predominant in ZIKV research. Global Ebola response and the United Nations Development Programme have indicated that more than 50 nations have contributed to Ebola response and funding much like the 48 nations highlighted in this article to have financed ZIKV research [8,9].

Like outbreaks such as that seen with Ebola, funding for ZIKV has been received from national governments, public and private sectors as well as internationally. ZIKV funding in this article comes from mainly public institutions such as The National Institute of Health (NIH) in the US, National Council for Scientific and Technological Development (CNPq) of Brazil and Institut Pasteur of France. These findings are analogous with those found in the article investigating the top 10 largest research funders which states that 40% of all health funding in high income countries comes from public and philanthropic organizations [10]. This information raises the question as to what barriers are preventing private, non-profits and NGOs from supporting ZIKV research or are these institutions supplying funds to investigations that are not being published. Additional research can therefore be conducted in future to answer this question and illuminate such hindrances. Other funders include United Kingdom Medical Research Council ($1.3 billion), European Commission ($3.7 billion),Wellcome Trust ($909.1 million) and USAID ($186.4 million) who are all mentioned as funders in the articles included in this review (**S2 Appendix**).

Brazil like other LAC is at the heart of the epidemic and as such research in these areas is of the greatest importance [11]. It is thus, fitting that Brazil was found to be one of the dominating nations contributing to the research in the included studies of this article. Of the 73 studies (50/268 +23/268) produced in LAC regions, Brazil accounts for 68.5% of the researching capacity. Other LAC countries exclusive of Brazil however, account for only 8.6% of the research. Similar trends are seen in other investigations which have highlighted Brazil as the dominant producer of randomized control trials in LAC and produces 70% of the research [12]. This revelation brings into question the factors that are contributing to the low volume of research in these areas. This data also makes it interesting to consider whether these areas are producing research that is possibly not being published.

In October of 2016 the European Commission announced two consortia—ZIKAlliance and ZikaPLAN—will receive €23M in financial assistance between them [13]. These two consortia are partnered with and coordinated by organizations affiliated with high income countries such as France, Sweden, United Kingdom and Brazil. These high economic countries have already been found in this review to receive most of the funding while low income countries that may benefit from the financial incentives continue to receive little financing. Brazil is heavily affected by the virus but other LAC countries such as Haiti or Honduras (low income countries) have received minimal frequency of funding [14]. The question posed here is, are there more instances where countries are willing to and have the research capacity, but do not have the financial means with which to conduct research.

As expected in situations involving disease where little in known, case series and case reports will be frequently utilized to gather and enhance the information base and these trends are seen in this article. At this stage this continued effort is relevant but there is also a need for the scientific community to increase frequency of investigations and methodologies, such as but not limited to laboratory based and longitudinal studies, to propose and test new hypotheses. Furthermore, investigations into the virus, vectors and reservoirs are also found to be dominant in the included studies, while public health interventions and causality research seem to be lagging behind. Awareness of these incidences will hopefully give the scientific community the momentum to look into aiming investigation into these lesser focused areas.

The findings in this article can be used to make suggestions for improvements in the researching endeavors of the scientific community. Since 38.1% (102/268) of the included studies did not report funding, it is important that authors consider supplying this information for future outbreaks. Secondly, it is also important for funders to indicate areas where they support research and their rationale for doing so. The investigation into the top 10 research funders mentions this information as not being readily available by some financing institutions [10]. Finally, there should be more published data giving details on funding for outbreaks such as ZIKV. The United nations has, up until January 2016, provided such information and has indicated the amount of money pledged, amount received and by whom and a list of recipients of these funds [15]

In conclusion, the data in this article suggests there is clearly an international effort into producing and funding research into the ZIKV. The data also implies a need for the readiness of accurate research funding information which will allow for a better regional understanding of the present funding situation. Collaborated and synchronized priorities need to be set so as to prevent research duplication and research concentration in one specific area. This will allow more effective and efficient research decisions with the optimum use of the finite resources available to researching bodies. Maximum allocation allows for more resources available to countries and institutions that presently desire financial assistance to make their contribution to the case of Zika transmission prevent and potential development of vaccines.

## Limitations

This article looked and how often funding institutions provided financial backing for ZIKV research. It did not however investigate the specific monetary value added to the research by individual bodies. For instance, some institutions may have well contributed more frequently than others, but some could have contributed less frequently but overall provided more funds to research. This review also only included articles that were available in English language. Excluding articles published in other languages could have eliminated studies that may have altered the results presented here. Lastly, this study did not statistically evaluate the relationship between type of funding and other categories (study type, study design). The frequencies in these categories suggest that meaningful relationships cannot be statistically drawn.

## Supporting information

S1 Appendix Characteristics of studies included in the review of Zika virus research funding. (XLS)

S2 Appendix Frequency distribution of funding institutions responsible for funding Zika virus research. (DOC)
