# Supplementary material for: An overview of financial sources being utilized to support Zika Virus published research
Source: PLoS One. 2017 Aug 17;12(8):e0183134. doi: 10.1371/journal.pone.0183134 (PMC5560720; doi:10.1371/journal.pone.0183134)
Supplement: S2 Appendix — (DOC) [file pone.0183134.s002.doc]

**S2 Appendix. Frequency distribution of funding institutions responsible for funding Zika Virus r**esearch

| **Funding institutions represented in included articles** | **Frequency** | **Percentage (%)** |
| --- | --- | --- |
| Abbott Laboratories | 2 | 0.6 |
| American Cancer Society | 1 | 0.3 |
| American Society of Tropical Medicine and Hygiene | 1 | 0.3 |
| Applied Research Fund María Viñas | 1 | 0.3 |
| Arizona State University | 1 | 0.3 |
| Autonomous Province of Trento | 1 | 0.3 |
| AXA Research Fund | 1 | 0.3 |
| Bart McLean Fund for Neuroimmunology Research | 1 | 0.3 |
| Baylor College of Medicine (BCM) | 1 | 0.3 |
| Beijing Nova Program | 1 | 0.3 |
| Bioinformatics Centre and Biochemistry, Mahatma Gandhi Institute of Medical Sciences | 1 | 0.3 |
| Brain and Behavior Research Foundation | 1 | 0.3 |
| Brazilian Development Bank | 1 | 0.3 |
| Burroughs Welcome Fund | 1 | 0.3 |
| Canadian Institutes of Health Research Fellowship | 1 | 0.3 |
| Centers for Disease Control and Prevention (CDC) | 4 | 1.3 |
| Cerus Corporation | 1 | 0.3 |
| Chinese Academy of Medical Sciences. | 1 | 0.3 |
| Chinese Academy of Sciences (CAS) | 3 | 1.0 |
| Connecticut Innovations’ Regenerative Medicine Research Fund | 1 | 0.3 |
| Consultancy Service for Enhancing Laboratory Surveillance of Emerging Infectious Disease of the Department of Health, Ho | 1 | 0.3 |
| Contrat de projets 2012–2014 | 1 | 0.3 |
| Croucher Senior Medical Research Fellowship | 1 | 0.3 |
| Czech Science Foundation | 1 | 0.3 |
| Defense Threat Reduction Agency (DTRA) | 3 | 1.0 |
| Dengue research Framework for Resisting Epidemics in Europe (DENFREE) | 3 | 1.0 |
| Department of Homeland Security | 1 | 0.3 |
| Deutsche Forschungsgemeinschaft (DFG). German Research Foundation | 1 | 0.3 |
| DeutscherAkademischerAustauschdienst (DAAD) | 1 | 0.3 |
| Development Program of Basic Sciences (PEDEClBA) | 1 | 0.3 |
| El Bosque University | 1 | 0.3 |
| Emory University | 1 | 0.3 |
| European Commission | 2 | 0.6 |
| European Union | 9 | 2.9 |
| European Virus Archive (EVA) | 2 | 0.6 |
| Federal University of São Paulo | 1 | 0.3 |
| Federated States of Micronesia Department of Health, Education and Social Affairs | 1 | 0.3 |
| Florida State Department | 1 | 0.3 |
| Florida State University | 2 | 0.6 |
| Foundation Carlos Chagas Filho Research Support of the State of Rio de Janeiro (FAPERJ) | 7 | 2.3 |
| Foundation Dormeur, Vaduz | 1 | 0.3 |
| Foundation of Research for the State of Minas Gerais (FAPEMIG) | 1 | 0.3 |
| Frederick National Laboratory for Cancer Research of the National Institute of Health | 1 | 0.3 |
| FreieUniversität Berlin (Free University of Berlin) | 1 | 0.3 |
| French Foreign Ministry | 1 | 0.3 |
| French Government | 4 | 1.3 |
| French Ministry of Health | 1 | 0.3 |
| Funding Authority for Studies and Projects (FINEP) | 1 | 0.3 |
| German Ministry of Health | 1 | 0.3 |
| Gorgas Memorial Institute for Health Studies Department of Research in Virology and Biotechnology | 1 | 0.3 |
| Guangzhou Science and Technology Program for Public Wellbeing | 1 | 0.3 |
| Helsinki University Hospital Funds | 1 | 0.3 |
| Henry M. Jackson Foundation | 1 | 0.3 |
| Howard Hughes Medical Institute | 1 | 0.3 |
| InfectiopôleSud Foundation | 1 | 0.3 |
| Infective Diseases Prevention and Cure Project of China | 1 | 0.3 |
| INSERM-transfert, France | 1 | 0.3 |
| Institut Pasteur | 10 | 3.2 |
| Internal Forensic and Scientific Services research and development project grant | 1 | 0.3 |
| Interuniversity Attraction Poles | 1 | 0.3 |
| Japan Agency for Medical Research and Development (AMED) | 2 | 0.6 |
| Japan Science and Technology Agency (JST) | 1 | 0.3 |
| Japanese Society for the Promotion of Science (JSPS) | 1 | 0.3 |
| Johns Hopkins Project Restore | 1 | 0.3 |
| Kansas State University | 1 | 0.3 |
| Kentucky Cabinet for Economic Development | 1 | 0.3 |
| Key Laboratory of Medical Molecular Virology, Fudan University | 1 | 0.3 |
| La Région Languedoc-Roussillon | 1 | 0.3 |
| Laboratory of Excellence for Epidemiology and Modeling (LExEM) | 1 | 0.3 |
| Leibniz Gemeinschaft | 1 | 0.3 |
| Mahidol University | 1 | 0.3 |
| March of Dimes | 2 | 0.6 |
| Maryland Stem Cell Research Fund | 1 | 0.3 |
| Massachusetts Institute of Technology (MIT) | 1 | 0.3 |
| Medical Research Council | 3 | 1.0 |
| Ministry of Education, Youth, and Sports of the Czech Republic | 1 | 0.3 |
| Ministry of Health of Singapore | 1 | 0.3 |
| Ministry of Research, Technology and Higher Education of the Republic of Indonesia | 1 | 0.3 |
| Ministry of Science and Higher Education, Poland | 1 | 0.3 |
| Ministry of Science and Technology, China | 8 | 2.6 |
| Mr Larry Chi-Kin Yung and Hui Hoy and Chow Sin Lan Charity Fund Limited | 1 | 0.3 |
| National Agency of Investigation (ANII) | 1 | 0.3 |
| National Basic Research Program of China | 1 | 0.3 |
| National Center for Global Health and Medicine (NCGM) | 1 | 0.3 |
| National Council for Scientific and Technological Development (CNPq) | 12 | 3.9 |
| National Institute for Health Research Biomedical Research Centre | 1 | 0.3 |
| National Institute of General Medical Sciences Models of Infectious Disease Agent Study | 1 | 0.3 |
| National Institute of Health (NIH) | 49 | 15.9 |
| National Key Project for Infectious Disease | 2 | 0.6 |
| National Key Technology Support Program | 1 | 0.3 |
| National Natural Science Foundation of China (NSFC) | 6 | 1.9 |
| National Research Agency of France (NRA) | 1 | 0.3 |
| National Research Foundation Investigatorship award | 1 | 0.3 |
| National Science Center | 1 | 0.3 |
| National Subvention for the Development of Research Organizations | 1 | 0.3 |
| Natural Environment Research Council (NERC) | 1 | 0.3 |
| Network of Cooperative Research in Tropical Diseases (RICET ) | 1 | 0.3 |
| New Caledonia Government | 2 | 0.6 |
| New York Stem Cell Foundation | 1 | 0.3 |
| Oswaldo Cruz Foundation (FIOCRUZ) | 3 | 1.0 |
| Pan American Health Organization (PAHO) | 2 | 0.6 |
| Panama Ministry of Economy and Finance | 1 | 0.3 |
| Pernambuco State Health Department | 1 | 0.3 |
| Prefeitura Municipal de Campina Grande/ Great Plains City Hall | 1 | 0.3 |
| Private Research supporter | 1 | 0.3 |
| Program for Zhejiang Leading Team of Science and Technology | 1 | 0.3 |
| Public Health Institute | 1 | 0.3 |
| Queensland Health | 2 | 0.6 |
| Ragon Institute of MGH, MIT and Harvard | 1 | 0.3 |
| REACTing through the ITMOs I3M and Neurosciences | 1 | 0.3 |
| Research on Emerging and Re-emerging Infectious Diseases by the Ministry of Health, Labor and Welfare, Japan | 1 | 0.3 |
| Royal Society and Leverhulme Trust Africa Award | 1 | 0.3 |
| Santa Maria Clinic | 1 | 0.3 |
| Sao Paulo Research Foundation (FAPESP) | 8 | 2.6 |
| Seacoast Biomedical Science Institute (SBSI) | 1 | 0.3 |
| Simons Foundation Autism Research Initiative (SFARI) | 2 | 0.6 |
| Sincelejo University Hospital | 1 | 0.3 |
| Singapore Ministry of Education | 1 | 0.3 |
| Stanford University | 1 | 0.3 |
| State Key Laboratory of Pathogen and Biosecurity, China | 3 | 1.0 |
| Strategic Research Theme Fund, The University of Hong Kong | 1 | 0.3 |
| Support Foundation of the State of Bahia Research (FAPESB) | 3 | 1.0 |
| Technological University of Pereira | 1 | 0.3 |
| Thailand Research Fund (TRF) | 2 | 0.6 |
| The Araucaria Support Scientific and Technological Development Paraná State Foundation (AF) | 2 | 0.6 |
| The Brazilian Ministry of Health | 3 | 1.0 |
| The Czech Science Foundation GA CR | 1 | 0.3 |
| The French National Research Agency (ANR) | 3 | 1.0 |
| The Higher Education Personnel Improvement Coordination (CAPES) of the Ministry of Education (MEC) | 7 | 2.3 |
| The International Medical Research Centre of Franceville (CIRMF) | 1 | 0.3 |
| The Ministry of Environment and Water Resources | 2 | 0.6 |
| The National Agency of Research | 1 | 0.3 |
| The National Major Special Program of Science and Technology of China | 1 | 0.3 |
| The Support Foundation Science and Technology of the State of Pernambuco (FACEPE) | 1 | 0.3 |
| The University of Texas | 1 | 0.3 |
| Total Gabon | 1 | 0.3 |
| UK Medical Research Council | 1 | 0.3 |
| United States Government | 1 | 0.3 |
| United States Public Health Service (USPHS) Institutional Research Training Award | 1 | 0.3 |
| University Hospital of Martinique | 1 | 0.3 |
| University of Florida | 1 | 0.3 |
| University of Pittsburgh Medical Center (UPMC) | 1 | 0.3 |
| University of Texas | 1 | 0.3 |
| University of Valle | 1 | 0.3 |
| University of Washington | 1 | 0.3 |
| University of Wisconsin–Madison | 1 | 0.3 |
| US Agency for International Development (USAID) | 4 | 1.3 |
| US Department of Defense Global Emerging Infections Surveillance and Response System | 3 | 1.0 |
| Vision Institute (IPEPO) | 1 | 0.3 |
| Washington University | 1 | 0.3 |
| Wellcome Trust | 3 | 1.0 |
| Wyss Institute for Biologically Inspired Engineering | 1 | 0.3 |
| Yap State Department of Health Service | 1 | 0.3 |
| Zhejiang Provincial Program for the Cultivation of High-level Innovative Health Talents | 1 | 0.3 |
| Zika Prevention and Control Project of China | 1 | 0.3 |
| Zika Special Project of the National Infectious Disease Control S and T Grand Project | 1 | 0.3 |
| **Total** | **308** | **100.0** |
